# Atorvastatin lowers ^68^Ga-DOTATATE uptake in coronary arteries, bone marrow and spleen in individuals with type 2 diabetes

**DOI:** 10.1007/s00125-023-05990-9

**Published:** 2023-08-15

**Authors:** Reindert F. Oostveen, Yannick Kaiser, Mia R. Ståhle, Nick S. Nurmohamed, Evangelos Tzolos, Marc R. Dweck, Jeffrey Kroon, Andrew J. Murphy, Damini Dey, Piotr J. Slomka, Hein J. Verberne, Erik S. G. Stroes, Nordin M. J. Hanssen

**Affiliations:** 1 Department of Vascular Medicine, Amsterdam Cardiovascular Sciences, Amsterdam UMC, University of Amsterdam, Amsterdam, the Netherlands; 2 Department of Experimental Vascular Medicine, Amsterdam Cardiovascular Sciences, Amsterdam UMC, University of Amsterdam, Amsterdam, the Netherlands; 3 Department of Cardiology, Amsterdam Cardiovascular Sciences, Amsterdam UMC, Free University, Amsterdam, the Netherlands; 4 British Heart Foundation Centre for Cardiovascular Science, University of Edinburgh, Edinburgh, UK; 5 Laboratory of Angiogenesis and Vascular Metabolism, VIB-KU Leuven Center for Cancer Biology, VIB, Leuven, Belgium; 6 Laboratory of Angiogenesis and Vascular Metabolism, Department of Oncology, KU Leuven and Leuven Cancer Institute (LKI), Leuven, Belgium; 7 Baker Heart and Diabetes Institute, Melbourne, VIC Australia; 8 Departments of Biomedical Sciences and Medicine, Cedars-Sinai Medical Center, Biomedical Imaging Research Institute, Los Angeles, CA USA; 9 Department of Radiology & Nuclear Medicine, Amsterdam UMC, University of Amsterdam, Amsterdam, the Netherlands

**Keywords:** Atherosclerosis, Inflammation, Macrophages, Molecular imaging, Statin

## Abstract

**Aims/hypothesis:**

Inflammation is a core component of residual cardiovascular risk in type 2 diabetes. With new anti-inflammatory therapeutics entering the field, accurate markers to evaluate their effectiveness in reducing cardiovascular disease are paramount. Gallium-68-labelled DOTATATE (^68^Ga-DOTATATE) has recently been proposed as a more specific marker of arterial wall inflammation than ^18^F-fluorodeoxyglucose (^18^F-FDG). This study set out to investigate whether ^68^Ga-DOTATATE uptake is amenable to therapeutic intervention in individuals with type 2 diabetes.

**Methods:**

Individuals aged >50 years with type 2 diabetes underwent ^68^Ga-DOTATATE positron emission tomography (PET)/computed tomography (CT) at baseline and after 3 months treatment with atorvastatin 40 mg once daily. Primary outcome was the difference in coronary ^68^Ga-DOTATATE uptake, expressed as target-to-background ratio (TBR). The secondary outcome was difference in bone marrow and splenic uptake, expressed as the standardised uptake value (SUV).

**Results:**

Twenty-two individuals with type 2 diabetes (mean age 63.2±6.4 years, 82% male, LDL-cholesterol 3.42±0.81 mmol/l, HbA_1c_ 55±12 mmol/mol [7.2%±3.2%]) completed both ^68^Ga-DOTATATE PET/CT scans. The maximum TBR was −31% (95% CI −50, −12) lower in the coronary arteries, and bone marrow and splenic ^68^Ga-DOTATATE uptake was also significantly lower post statin treatment, with a mean percentage reduction of −15% (95% CI −27, −4) and −17% (95% CI −32, −2), respectively.

**Conclusions/interpretation:**

^68^Ga-DOTATATE uptake across the cardio–haematopoietic axis was lower after statin therapy in individuals with type 2 diabetes. Therefore, ^68^Ga-DOTATATE is promising as a metric for vascular and haematopoietic inflammation in intervention studies using anti-inflammatory therapeutics in individuals with type 2 diabetes.

**Trial registration:**

ClinicalTrials.gov NCT05730634

**Graphical Abstract:**

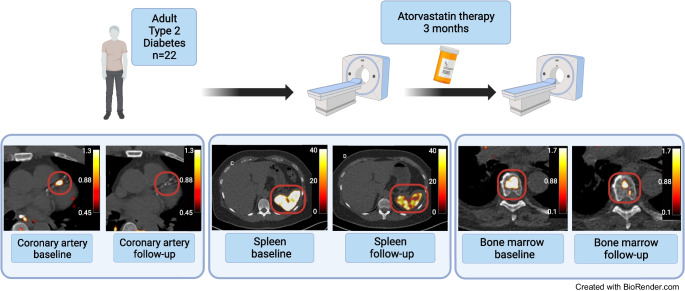

**Supplementary Information:**

The online version contains peer-reviewed but unedited supplementary material available at 10.1007/s00125-023-05990-9.


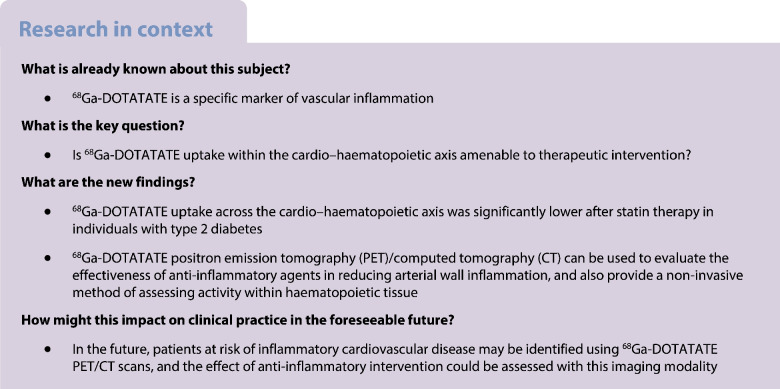


## Introduction

Type 2 diabetes is hallmarked by systemic inflammation [1], which is a pivotal process driving atherogenesis [2]. Specifically, diabetes is associated with activation of the cardio–haematopoietic axis [3], wherein inflammatory monocytes produced by the haematopoietic organs migrate to atherosclerotic plaques, accelerating atherosclerotic disease. With new anti-inflammatory therapeutics, such as ziltivekimab [4], entering the clinical stage of testing, accurate surrogate markers of vascular inflammation that reflect activation of the cardio–haematopoietic axis are needed to prevent large scale exposure to ineffective immunosuppressive drugs. A highly promising tool to meet this end is Gallium-68-labelled [1,4,7,10-tetraazacyclododecane-*N*,*N*',*N*'',*N*'''-tetraacetic acid]-d-Phe1, Tyr3-octreotate (^68^Ga-DOTATATE), a positron emission tomography (PET) tracer with high affinity for the somatostatin type 2 receptor (SSTR2) that is highly expressed in activated (M1) macrophages within atherosclerotic plaques [5]. A crucial issue for surrogate imaging markers is the amenability of the signal towards therapeutic interventions. In the present study, we sought to investigate whether statin treatment, an established intervention to reduce cardiovascular events and with anti-inflammatory activity [6] is able to reduce ^68^Ga-DOTATATE uptake in the coronary arteries and aorta, and in the bone marrow and spleen as the key haematopoietic organs.

## Methods

Detailed methods are included in the electronic supplementary material (ESM).

In short, individuals with type 2 diabetes from the Amsterdam UMC were eligible, if they were >50 years old, statin-naive for at least 6 weeks, had HbA_1c_ levels <65 mmol/mol (8.1%) and no changes in glucose-lowering medication within 3 months of inclusion. All patients provided written informed consent. Atorvastatin 40 mg once daily was initiated after the first ^68^Ga-DOTATATE PET/computed tomography (CT) scan, for a period of 3 months. After statin therapy was completed, the patients were subjected to a follow-up ^68^Ga-DOTATATE PET/CT scan. Blood was collected at baseline and follow-up visits, to determine lipid, metabolic and inflammatory variables. To quantify uptake of ^68^Ga-DOTATATE in coronary arteries, we used the maximum target-to-background ratio (TBR_max_). We reported both the per vessel TBR_max_, as well as the overall coronary tree TBR_max_. The maximum standardised uptake value (SUV_max_) in bone marrow and spleen was assessed by drawing volumes of interest (VOIs) around each respective structure. Methods regarding the measurement of uptake in lung and muscle tissue can be found in the ESM Methods. The study protocol was approved by the local medical ethics committee and performed in accordance with the Declaration of Helsinki

## Results

### Patient characteristics

Of the 24 patients included, one patient withdrew from the study prior to first scan and another patient discontinued study participation owing to myalgia and did not complete follow-up PET/CT. Accordingly, 22 patients (mean age 63±6 years, 82% male, HbA_1c_ 55 mmol/mol [7.2%]) were included in subsequent analyses. A flowchart of the inclusions can be found in ESM Fig. 1. The baseline and follow-up characteristics after 12 weeks of statin therapy are listed in Table 1. Of note, LDL-cholesterol levels decreased by 58% and C-reactive protein (CRP) by 20%, while the HbA_1c_ did not decrease at follow-up.

**Table 1:** Baseline characteristics and changes in laboratory variables and imaging parameters after 12 weeks of atorvastatin treatment

Study participant characteristics (*n*=22)	Baseline	Follow-up	Percentage difference at follow-up	*p* value
Age, years	63.2±6.4	–	–	–
Male sex	18 (81.8)	–	–	–
Smoking				
Never	5 (22.7)	–	–	–
Former	16 (72.7)	–	–	–
Active	1 (4.5)	–	–	–
BMI, kg/m^2^	29.1±3.8	–	–	–
Systolic blood pressure, mmHg	140±16	–	–	–
Diastolic blood pressure, mmHg	86±8	–	–	–
Laboratory variables				
CRP, mg/l	1.25 [0.80, 2.28]	0.90 [0.60, 2.00]	−20 [–33, 0]	0.077
Fasting glucose, mmol/l	8.4±2.1	8.82±2.92	−5 (−9, 18)	0.493
HbA_1c_, mmol/mol	55±12	58±14	6 (−0.9, 12)	0.087
HbA_1c_, %	7.2±3.2	7.5±3.4	6 (−0.9, 12)	0.087
Total cholesterol, mmol/l	5.66±1.01	3.36±0.97	−41 (−44, −37)	<0.001
HDL-cholesterol, mmol/l	1.20±0.33	1.19±0.35	1 (−5, 3)	0.530
LDL-cholesterol, mmol/l	3.42±0.81	1.44±0.62	−58 (−65, −51)	<0.001
Triglycerides, mmol/l	1.99 [1.08, 2.65]	1.16 [0.80, 1.61]	−32 [−48, −19]	<0.001
Apolipoprotein B, g/l	1.13±0.21	0.61±0.22	−46 (−51, −41)	<0.001
Imaging parameters				
Coronary artery calcium, AU	378.70 [56.57, 700.80]	337.30 [56.85, 737.52]	2 [−12, 20]	0.411
Highest TBR_max_ in coronary arteries	2.27±0.91	1.57±0.39	−31 (−50, −12)	<0.05
Left anterior descending coronary artery (SUV_max_)	1.17±0.35	1.05±0.33	−7 (−22, 7)	0.304
Left anterior descending coronary artery (TBR_max_)	1.82±0.66	1.44±0.39	−21 (−38, −3)	<0.05
Left circumflex coronary artery (SUV_max_)	1.07±0.32	1.07±0.41	−0.3 (−14, 14)	0.955
Left circumflex coronary artery (TBR_max_)	1.82±0.66	1.43±0.43	−21 (−37, −6)	<0.05
Right coronary artery (SUV_max_)	1.20±0.70	1.20±0.70	−23 (−57, 10)	0.163
Right coronary artery (TBR_max_)	1.89±0.94	1.30±0.21	−31 (−55, −8)	<0.05
Ascending aorta (SUV_max_)	1.84±0.45	1.53±0.51	−15 (−30, 0.3)	0.054
Ascending aorta (TBR_max_)	2.90±0.99	1.89±0.88	−25 (−45, −6)	<0.05
Bone marrow (SUV_max_)	1.99±0.55	1.69±0.45	−15 (−27, −4)	<0.05
Spleen (SUV_max_)	37.9±14.0	31.32± 6.88	−17 (−32, −2)	<0.05
Background uptake in left atrium (SUV_max_)	0.67±0.22	0.74±0.22	10 (−6, 28)	0.194
Lung (SUV_mean_)	0.23±0.07	0.23±0.06	−0.6 (−9, 10)	0.897
Lung (TBR_mean_)	0.36±0.09	0.33±0.10	−9 (−25, 7)	0.230
Muscle (SUV_mean_)	0.31±0.10	0.34±0.09	8 (−3, 20)	0.150
Muscle (TBR_mean_)	0.51±0.17	0.50±0.15	−2 (−22, 18)	0.829

Values are presented as mean ± SD, median [IQR] or number (percentage). Differences are depicted as mean (95% CI) or median [IQR] percentage difference

AU, Agatston units

### Changes in ^68^Ga-DOTATATE uptake in the cardio–haematopoietic axis after atorvastatin treatment

**Fig. 1:**
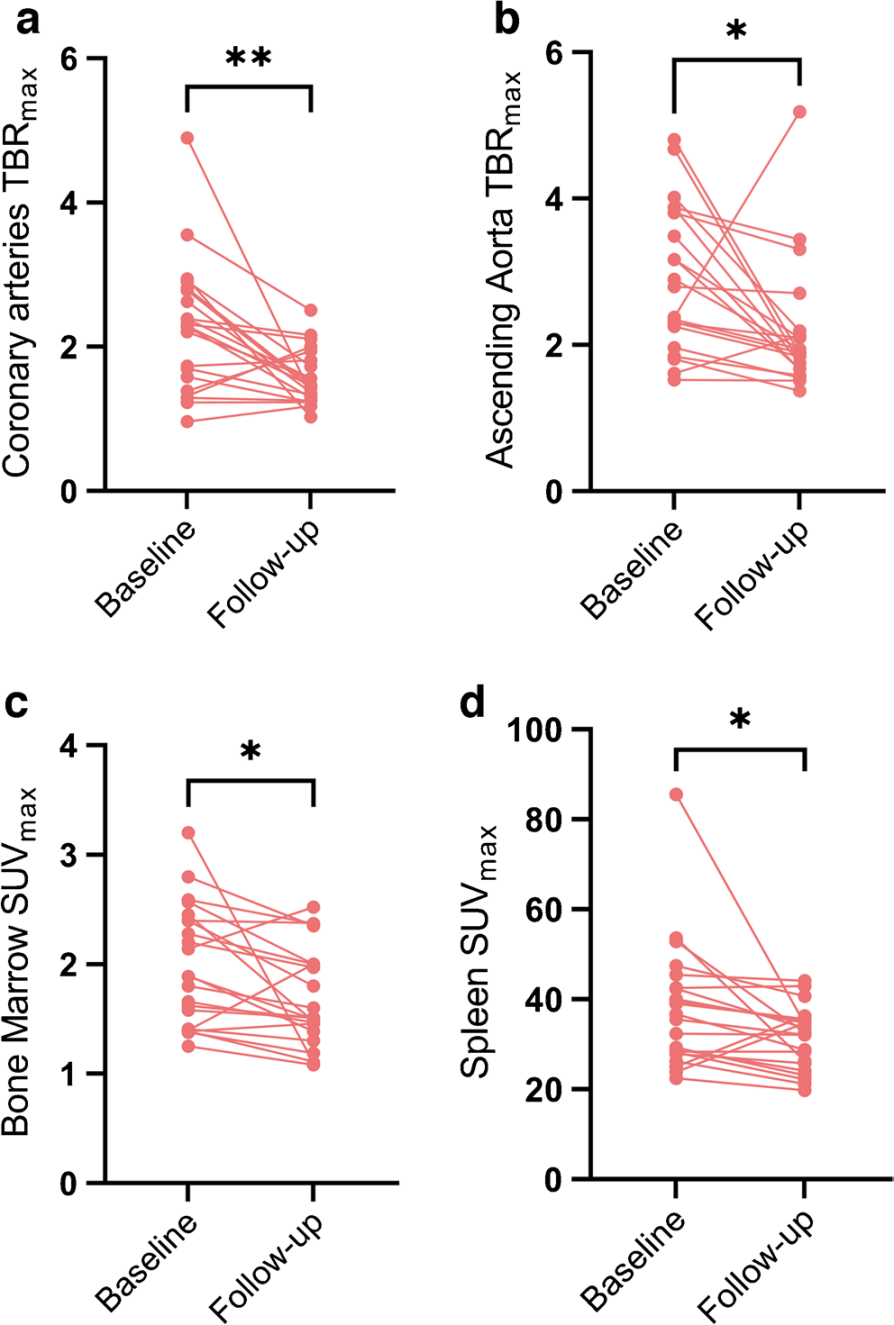
^68^Ga-DOTATATE uptake throughout the cardio–haematopoietic axis at baseline and follow-up. The uptake of the ^68^Ga-DOTATATE within the coronary arteries, ascending aorta, bone marrow and spleen, at baseline and after 12 weeks of atorvastatin treatment in patients with type 2 diabetes. Paired *t* tests were performed to test for statistical significance: *p<0.05, **p<0.01

The imaging parameters at baseline and follow-up are shown in Table 1. Overall, we observed a consistent and significant decrease of ^68^Ga-DOTATATE TBR_max_ in the coronary arteries (−31%) and ascending aorta (−25%), and SUV_max_ in bone marrow (−15%) and spleen (−17%) (Fig. 1 and Table 1). The difference in TBR_max_ within the ascending aorta correlated with the difference in ^68^Ga-DOTATATE within the coronary arteries (*r*=0.49, *p*=0.025). In contrast, no correlations were found between changes in CRP (*r*=0.24, *p*=0.289) or LDL-cholesterol levels (*r*=0.214, *p*=0.349) with changes in coronary ^68^Ga-DOTATATE.

## Discussion

We provide evidence of therapeutic modulation of vascular ^68^Ga-DOTATATE uptake in individuals with type 2 diabetes. We discovered significant reductions in ^68^Ga-DOTATATE uptake in the coronary arteries and ascending aorta after a 12 week regimen of atorvastatin 40 mg daily (Fig. 1). These changes did not correlate with CRP, which currently is the most used surrogate marker of vascular inflammation. Interestingly, ^68^Ga-DOTATATE uptake in haematopoietic organs was also reduced substantially, suggesting that ^68^Ga-DOTATATE PET/CT could be used to assess inflammation in other key organs that contribute to atherosclerotic cardiovascular disease. Collectively, these data show that ^68^Ga-DOTATATE PET/CT holds promise as a surrogate marker to non-invasively evaluate the treatment response of inflammatory activity throughout the cardio–haematopoietic axis in individuals with type 2 diabetes.

We observed a significant change in the coronary TBR_max_ after statin treatment, indicative of a reduction in inflammatory activity in the coronary arteries [5]. We substantiated this finding by demonstrating a similar effect in the ascending aorta, while the background uptake of ^68^Ga-DOTATATE measured in the left atrium did not change after statin treatment. Comparing this imaging modality with the currently used ^18^F-fluorodeoxyglucose (^18^F-FDG) PET/CT, not only can ^68^Ga-DOTATATE readily be used to evaluate vascular inflammation in the coronary arteries without being affected by myocardial spillover, but also greater effect sizes are observable. This allows future studies to have smaller study populations when using ^68^Ga-DOTATATE instead of ^18^F-FDG. A previous study examining changes in arterial ^18^F-FDG uptake after 12 weeks of atorvastatin treatment reported a mean reduction of 14.4% in TBR_max_ [7], whereas our study demonstrated a mean reduction of 31% in ^68^Ga-DOTATATE TBR_max_.

We observed a notably higher uptake of ^68^Ga-DOTATATE in the bone marrow and spleen in individuals with type 2 diabetes, at a similar background signal as was previously reported as physiological uptake in apparently healthy non-diabetic individuals [8]. Notably, we also identified a lower ^68^Ga-DOTATATE uptake within the bone marrow and spleen after statin treatment. Future studies, including bone marrow biopsies in tandem with ^68^Ga-DOTATATE PET/CTs, are required to determine whether a decrease in ^68^Ga-DOTATATE uptake is caused by a decreased production of M1 macrophages, polarisation towards an M2 phenotype, or a combination of the two. In mice both chronic and transient intermittent hyperglycaemia promote myelopoiesis [3]. The fact that ^68^Ga-DOTATATE uptake reduction in the haematopoietic organs bears striking resemblance to a reduction in the coronary arteries is in accordance with the hypothesis that haematopoietic activation may be a contributing factor for atherosclerosis in individuals with type 2 diabetes. In support of this, ^18^F-FDG studies have demonstrated that haematopoietic uptake in apparently healthy individuals is also associated with early atherosclerosis [9].

Limitations of our study include the lack of a placebo group. Since international guidelines recommend statin therapy for all patients with diabetes mellitus of 40 years and older [10], it was not considered ethical to include a placebo group. Nonetheless, the interpretation of our results is unlikely to be affected by lack of a control group, as we do not consider spontaneous resolution of atherosclerotic inflammation to be a likely phenomenon. Second, we may have underestimated the total coronary plaque burden because vascular PET imaging has relatively low spatial resolution. Therefore, to limit the challenges of identifying the coronary arteries, we performed ECG-gated, breathing-corrected PET/CT scans. Accordingly, we drew VOIs along the grooves of the coronary tree, to approximate the TBR_max_ of the coronary arteries as closely as possible. Despite the spatial limitations inherent to current PET/CT techniques, we were clearly able to detect a strong decrease in coronary uptake of ^68^Ga-DOTATATE.

In conclusion, we show that ^68^Ga-DOTATATE PET can be used to identify changes in arterial wall inflammation, as well as activity in haematopoietic organs, providing a rapid readout after just 3 months of drug therapy. Therefore, ^68^Ga-DOTATATE holds promise as a surrogate marker in upcoming intervention trials that dampen inflammation. However, owing to limitations of the study design, these results will require further confirmation.

### Supplementary Information

Below is the link to the electronic supplementary material.

Supplementary file1 (PDF 255 KB)

## Data Availability

The data that support the findings of this study are not openly available due to reasons of sensitivity and are available from the corresponding author upon reasonable request. Data are located in controlled access data storage at the Amsterdam UMC, location AMC.

## Abbreviations

CRP: C-reactive protein
CT: Computed tomography
FDG: Fluorodeoxyglucose
PET: Positron emission tomography
SUV: Standardised uptake value
TBR: Target-to-background ratio
VOI: Volume of interest
