# Supplementary material for: Atorvastatin lowers 68Ga-DOTATATE uptake in coronary arteries, bone marrow and spleen in individuals with type 2 diabetes
Source: Diabetologia. 2023 Aug 15;66(11):2164–9. doi: 10.1007/s00125-023-05990-9 (PMC10542709; doi:10.1007/s00125-023-05990-9)
Supplement: Supplementary file 1 — Supplementary file1 (PDF 255 KB) [file 125_2023_5990_MOESM1_ESM.pdf]

Electronic Supplemental Materials (ESM)

Methods

Study procedures

Patients were recruited from outpatient clinic consultations for long term management of type 2 diabetes mellitus. Patients were eligible for inclusion if they were over the age of 50, and had type 2 diabetes with HbA1c levels <65 mmol/mol (8.1%). They also had to be statin-naïve or willing to halt their statin therapy for a period of at least six weeks, and no changes in anti-diabetic medication within three months of inclusion were allowed. Patients with a history of overt cardiovascular disease were excluded. Race and ethnicity data were not collected, since analyses of this data were outside the scope of this study's sample size. All patients provided written informed consent. Atorvastatin 40mg once daily would be initiated after the first ^{68}Ga-DOTATATE PET/CT, for a period of 12 weeks. After statin therapy was completed, the patients were subjected to a follow-up ^{68}Ga-DOTATATE PET/CT. The study protocol was approved by the local medical ethics committee and performed in accordance with the Declaration of Helsinki.

Laboratory measurements

Blood was collected at baseline and follow-up visits, to determine lipid, metabolic and inflammatory parameters. C-reactive protein (CRP), HbA1c, fasting glucose, total cholesterol, triglyceride, and high-density-lipoprotein cholesterol (HDL-C) and apolipoprotein B (ApoB) levels were measured. Low-density-lipoprotein cholesterol (LDL-C) was calculated using the Friedewald formula [1].

Image acquisition and reconstruction

PET/CT image acquisition was performed 60 minutes after intravenous administration of approximately 100MBq ^{68}Ga-DOTATATE, a Biograph mCT Flow PET/CT scanner (Siemens, Germany) equipped with enhanced axial field of view (TrueV) was used. First, a low dose CT scan was performed from skull base to spleen followed by a PET scan continuous bed motion at 1mm/s. CT data was used for PET attenuation correction and PET data were reconstructed with TrueX algorithm [2] in 4mm×4mm×5mm voxels.

Then a sequential coronary artery calcium (CAC) scan was performed using an end-inspiratory breath-hold with prospective electrocardiogram (ECG) gating, tube voltage 120kVp, and tube current 60mAs. Thereafter, a prospective ECG gated PET scan of the heart was performed for 20 minutes. PET Data were reconstructed in mid-diastolic phase, with a phase length of 25% using the same settings as described above, attenuation correction was performed using the first low dose CT.

Image analysis

Image analyses were performed on a dedicated workstation (FusionQuant) and analysed by an experienced observer (ET). Analyses of the follow-up scans were performed six weeks after the baseline analysis. To quantify uptake of ^{68}Ga-DOTATATE in coronary arteries, we used the maximum target to background ratio (TBR_{max}) due to extensive experience with this method of measuring tracer uptake within the arterial wall [3]. The administered activity always varies slightly between scans due to a multitude of factors, primarily the time delay between the labelling of the tracer and when the tracer is administered to the patient. Therefore, to correct for the difference in administered activity, it is standard practice to correct the uptake of tracer in the coronary arteries for the uptake in the blood pool. The TBR_{max} was calculated by dividing the standardized uptake value (SUV) of target tissue by background SUV_{mean}. We measured the SUV_{max} in the coronary arteries by drawing volumes of interest (centrelines with

4mm diameter) across the main coronary arteries (left anterior descending (LAD), left circumflex (LCx) and right coronary artery (RCA)) starting from the ostium and then following the artery across the coronary groove. These encompass all the main epicardial coronary vessels and their immediate surroundings (4mm radius) facilitating per-vessel and per-patient uptake quantification. For this study we evaluated ^{68}Ga-DOTATATE activity along the entire course of the coronary arteries and we included the left main in the LAD volume of interest (VOI). Within such VOIs, we measured the whole vessel SUV_{max}, and used these to derive coronary TBR values after correction for blood pool. If scatter caused by the liver was identified near the RCA, the VOI would be adjusted to avoid these hotspots. We assessed the SUV_{mean} ^{68}Ga-DOTATATE uptake in the left atrium by using a cylinder 10 mm in diameter and height. CAC measurements were performed according to the Agatston method [4]. Finally, ascending aorta uptake was calculated by drawing VOI starting from the sinotubular junction and finishing immediately proximal to the junction with the brachiocephalic artery [5]. The final diameter of the region of interest around the aorta was equal to the maximal luminal diameter of the aorta of that section plus 4mm (the approximate spatial resolution of PET).

SUV_{max} in bone marrow and spleen was assessed by drawing VOIs around each respective structure. For the spleen we used a cylinder of 10mm radius and 10mm height. We drew the VOI around the hottest point of the splenic uptake. For bone marrow uptake we drew VOI in the middle of the thoracic vertebrates and calculated the mean SUV_{max} value. SUV_{mean} in the lung was assessed by drawing a sphere 15mm in diameter in the left lung at the level of the pulmonary artery bifurcation. For the SUV_{mean} in muscle tissue we also drew a sphere 15mm in diameter in the left pectoral muscle at the level of the pulmonary artery bifurcation. We corrected these SUV_{mean} values for the blood pool to derive the TBR values.

Statistical Analysis

Our sample size (n=24) was chosen to detect a ≥12% reduction in coronary TBR_{max} after atorvastatin treatment, with 80% power and a two-sided p value of <0.05. This was based on previous studies which evaluated changes in ^{18}F-FDG uptake after atorvastatin 40mg treatment, since Tarkin et al. found a strong correlation between the uptake of ^{68}Ga-DOTATATE and ^{18}F-FDG in the coronary arteries and aorta [6,7]. All data are expressed as mean ± standard deviation, median [interquartile range] or number (percentage), as appropriate. The primary outcome of this study was TBR_{max} reduction in the coronary arteries. Secondary outcomes were the reduction in bone marrow and spleen SUV_{max}. Changes in normally distributed data were assessed using a paired t-test, whereas those between non-normally distributed data were assessed using the Paired Samples Wilcoxon Signed-Rank test. All changes are expressed as the percentage difference between baseline and follow-up. Finally, we tested correlations between ^{68}Ga-DOTATATE uptake, CAC, glucose, LDL-C, and CRP using the Pearson correlation coefficient or Spearman's rank correlation coefficient for normally and non-normally distributed data, respectively. Statistical analyses were performed using R version 4.0.3. Graphical representations of the data were created using GraphPad Prism version 9.4.0.

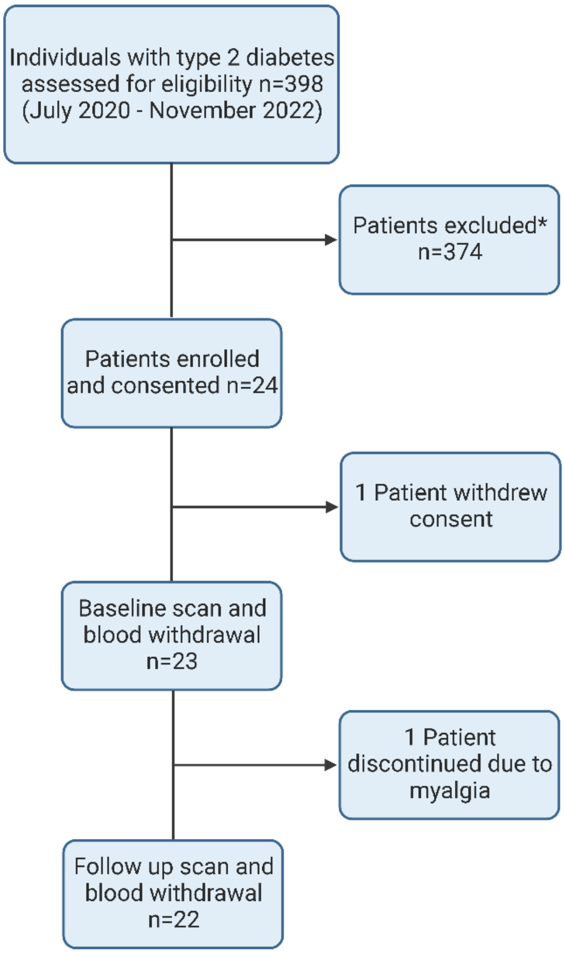

ESM Fig 1. Flowchart inclusions

References:

1. Friedewald WT, Levy RI, Fredrickson DS. Estimation of the concentration of low-density lipoprotein cholesterol in plasma, without use of the preparative ultracentrifuge. *Clin Chem*. 1972;18(6):499-502.

2. Knäusl B, Rausch IF, Bergmann H, Dudczak R, Hirtl A, Georg D. Influence of PET reconstruction parameters on the TrueX algorithm. A combined phantom and patient study. *Nuklearmedizin*. 2013;52(1):28-35. doi:10.3413/Nukmed-0523-12-07

3. van der Valk FM, Verweij SL, Zwinderman KAH, et al. Thresholds for Arterial Wall Inflammation Quantified by (18)F-FDG PET Imaging: Implications for Vascular Interventional Studies. *JACC Cardiovasc Imaging*. 2016;9(10):1198-1207. doi:10.1016/j.jcmg.2016.04.007

4. S. AA, R. JW, J. HF, R. ZN, Manuel V, Robert D. Quantification of coronary artery calcium using ultrafast computed tomography. *J Am Coll Cardiol*. 1990;15(4):827-832. doi:10.1016/0735-1097(90)90282-T

5. Fletcher AJ, Tew YY, Tzolos E, et al. Thoracic Aortic ^{18}F-Sodium Fluoride Activity and Ischemic Stroke in Patients With Established Cardiovascular Disease. *JACC Cardiovasc Imaging*. 2022;15(7):1274-1288. doi:10.1016/j.jcmg.2021.12.013

6. Tarkin JM, Joshi FR, Evans NR, et al. Detection of Atherosclerotic Inflammation by 68 Ga-DOTATATE PET Compared to [18 F]FDG PET Imaging. *J Am Coll Cardiol*. 2017;69(14):1774-1791. doi:10.1016/j.jacc.2017.01.060

7. van der Valk FM, Bernelot Moens SJ, Verweij SL, et al. Increased arterial wall inflammation in patients with ankylosing spondylitis is reduced by statin therapy. *Ann Rheum Dis*. 2016;75(10):1848-1851. doi:10.1136/annrheumdis-2016-209176
